# G-protein coupled receptor 64 is required for decidualization of endometrial stromal cells

**DOI:** 10.1038/s41598-017-05165-8

**Published:** 2017-07-10

**Authors:** Jung-Yoon Yoo, Jong Il Ahn, Tae Hoon Kim, Sungryul Yu, Ji Yeon Ahn, Jeong Mook Lim, Jae-Wook Jeong

**Affiliations:** 1 Deparment of Obstetrics and Gynecology & Reproductive Biology, Michigan State University, College of Human Medicine, Grand Rapid, MI 49503 United States; 2 Research Institutes of Agriculture and Life Sciences, Seoul National University, Seoul, 08826 Republic of Korea; 3 Department of Agricultural Biotechnology, Seoul National University, Seoul, 08826 Republic of Korea; 4 Department of Clinical Laboratory Science, Semyung University, Jecheon, 27136 Republic of Korea; 5 Department of Women’s Health, Spectrum Health System, Grand Rapids, MI 49341 United States

## Abstract

Although GPR64 has an important role for male fertility, its physiological roles in the female reproductive system are still unknown. In the present study, immunohistochemical analysis reveals a spatiotemporal expression of GPR64 in the uterus during early pregnancy. Observation of remarkable induction of GPR64 expression in uterine decidual cells points to its potential physiological significance on decidualization. The decidualization of uterine stromal cells is a key event in implantation. Progesterone (P4) signaling is crucial for the decidualization of the endometrial stromal cells for successful pregnancy. Therefore, we examined ovarian steroid hormone regulation of GPR64 expression in the murine uterus. P4 induced GPR64 expression in the epithelial and stromal cells of the uterus in ovariectomized wild-type mice, but not in PRKO mice. ChIP analysis confirmed that PGR proteins were recruited on progesterone response element of *Gpr64* gene in the uteri of wild-type mice treated with P4. Furthermore, the expression of *GPR64* was increased in human endometrial stromal cells (hESCs) during *in vitro* decidualization. Interestingly, small interfering RNA (siRNA)-mediated knockdown of *GPR64* in hESCs remarkably reduced decidualization. These results suggest that *Gpr64* has a crucial role in the decidualization of endometrial stromal cells.

## Introduction

Major functions of the uterus include receiving the embryo, sheltering the fetus during pregnancy, and delivering the newborn at term. The uterine endometrium consists of glandular and luminal epithelium, and stroma. During pregnancy, the uterus undergoes dynamic molecular and morphological changes to allow for embryo implantation and development. These changes of uterine components are tightly regulated by two ovarian steroid hormones, estrogen (E2) and progesterone (P4)^1^. The success of fertility is dependent on the balanced interaction of E2 and P4 acting through their receptors, E2 receptor (ESR) and P4 receptor (PGR). E2 is known to stimulate uterine epithelial cell proliferation while P4 is inhibitory to E2-mediated effects^2, 3^.

P4-PGR signaling is essential in the uterus for successful implantation, decidualization, and glandular development^4, 5^. P4 is critical for the development of decidual tissues, and if fertilization occurs, high circulating P4 levels are important not only for facilitating implantation, but also for maintaining pregnancy by stimulating uterine growth and opposing the actions of factors involved in myometrial contraction. Previous research using a transgenic mouse model with a null mutation in the *Pgr* gene (PRKO) demonstrates the critical role for PGR in P4-mediated uterine responses^5, 6^, and have led to the identification of several P4-PGR signaling pathways within the uterus^7^.

During early pregnancy, the uterine stromal cells undergo a process called decidualization. P4-PGR signaling is critical in the process of decidualization^8^. Decidualization is unique to species with hemochorial placenta, such as human, primates and rodents and serves to protect the maternal uterus during trophoblast invasion as well as providing nourishment to the embryo^9^. Endometrial stromal cells undergoing decidualization become plumper, acquire a secretory epithelioid-like morphology, and secrete a variety of factors, including prolactin (PRL) and insulin-like growth factor binding protein 1 (IGFBP1)^10^. Moreover, this transformation results in extensive changes in cellular gene expression, including alterations in steroid hormone receptor, extracellular matrix (ECM) and cytoskeletal gene profiles^11–13^. Multiple transgenic mouse models demonstrate that the decidualization process is important for the maintenance of pregnancy^5, 14–18^.

Decidualization is the P4 mediated differentiation of small stromal fibroblast into large epithelioid decidual cells. In humans, decidualization of the stromal compartment occurs in the mid-secretory phase of the menstrual cycle, independently of pregnancy^10^. The decidual reaction is inhibited in PGR-A knock-out mice, but not PGR-B knock-out mice, suggesting a critical of PGR-A in this process^19–21^. In humans, decidualization process occurs in stromal cells surrounding the spiral arties approximately 10 days after the postovulatory rise in ovarian P4 level, indicating that the expression of the decidua-specific genes is under the direct control of activated PGR. Therefore, the identification of P4-PGR regulated genes is crucial in understanding the causes of impairments in fertility.

G-protein coupled receptor 64 (GPR64) is also known as Adhesion g protein-coupled receptor G2 (ADGRG2) and Human Epididymis-specific protein 6 (HE6), and a member of the G protein-coupled receptor (GPCR) family described as an epididymis-specific transmembrane protein^22, 23^. GPCRs have a pivotal role in cancer development and progression^24, 25^. The levels of *GPR64* are significantly overexpressed in the Wnt signaling-dependent subgroup of medulloblastoma^26^ and higher in Ewing sarcomas^27^. GPR64 promotes tumor invasion and metastasis through induction of the placental growth factor (PGF) and metalloproteinase (MMP1) expression^27^. *GPR64* also suggests a novel target gene candidate in ovarian endometrioid adenocarcinoma caused by dysregulation of β-catenin/T-cell factor (TCF) signaling by using oligonucleotide microarrays^28^.

Furthermore, GPR64 is crucial for male fertility^29^. *Gpr64* knockout male mice result in infertility due to sperm stasis and duct obstruction by abnormal fluid reabsorption. Additionally, hemizygous knockout males and homozygous knockout females show no apparent developmental or behavioral abnormalities compared with wild-type littermates^29^. However, the role of *Gpr64* in the female reproductive tract is unclear. In this study, we explored the spatiotemporal expression profile and regulation of *Gpr64* in the response to P4-PGR and during early pregnancy in mice uteri. To investigate the function of *GPR64*, we used the well-characterized *in vitro* primary human endometrial stromal cell (hESC) decidualization model.

## Results

### The expression of Gpr64 in the mouse uterus during early pregnancy

**Figure 1:**
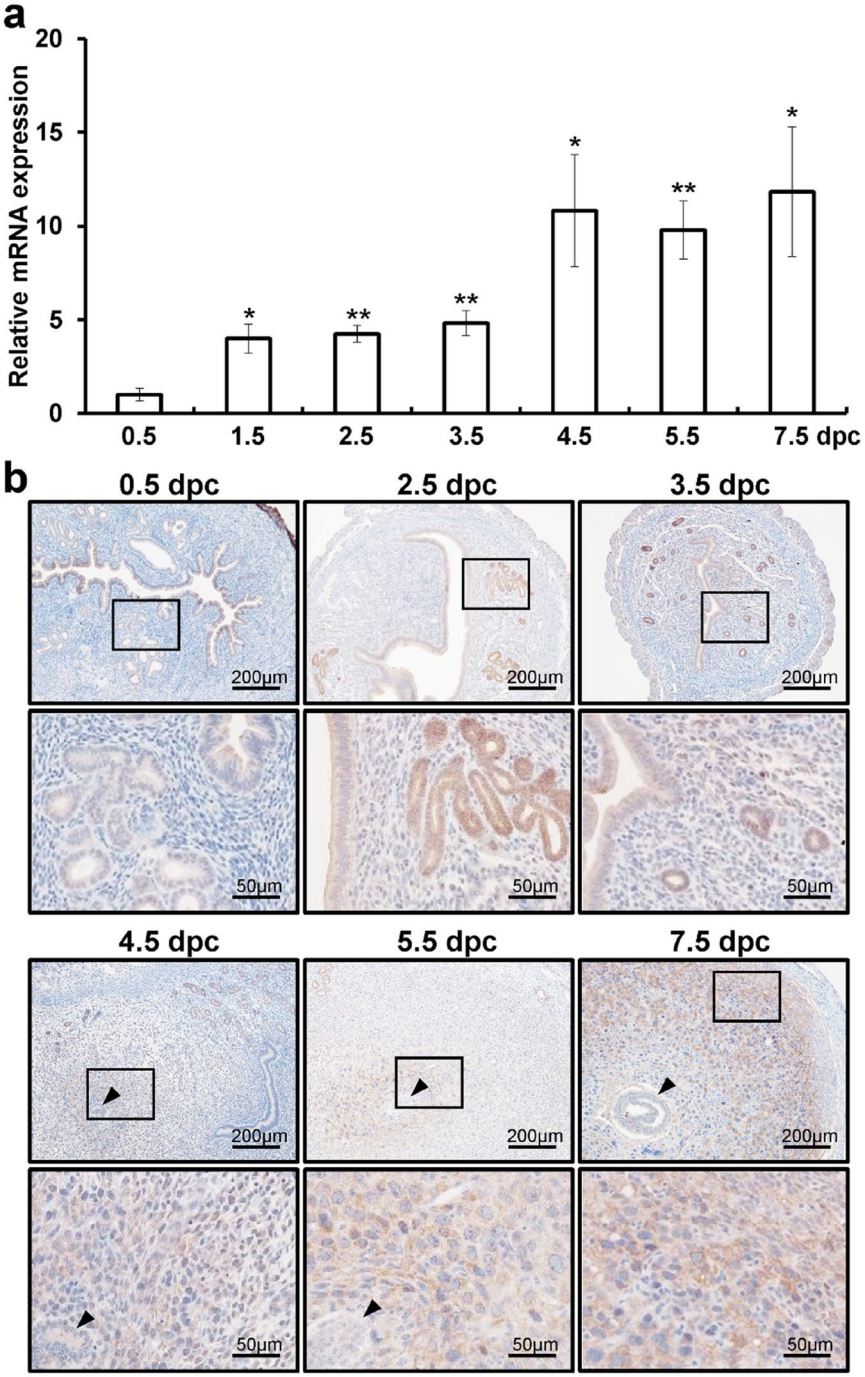
The expression patterns of *Gpr64* during early pregnancy. (**a**) The expression levels of *Gpr64* were measured in uteri during early pregnancy. Total RNA used for the quantitative real-time PCR assays was prepared from early pregnancy uteri. The results represent the mean ± SEM of three independent RNA sets. *p < 0.05 and **p < 0.01. (**b**) The immunohistochemistry analysis of GPR64 during natural pregnancy was investigated at 0.5 dpc, 2.5 dpc, 3.5 dpc, 4.5 dpc, 5.5 dpc, and 7.5 dpc. Black arrow head indicates embryo. Nuclei were counterstained with hematoxylin.

To investigate the expression profile of *Gpr64* during early pregnancy, we examined levels of *Gpr64* in wild-type female uteri during early pregnancy. The initiation of pregnancy was marked by the presence of a postcoital vaginal plug (0.5 dpc). The levels of *Gpr64* mRNA were detected on 0.5 dpc, which gradually increased until 7.5 dpc, reaching statistical significance after 1.5 dpc in the uterus (Fig. 1a). To further investigate the spatiotemporal expression profile of GPR64 protein during early pregnancy, we performed immunohistochemistry analysis for GPR64 (Fig. 1b). GPR64 proteins were very weak in the epithelium and stroma at 0.5 dpc. Consistent with the real-time PCR results, the levels of GPR64 were significantly higher detected in the glandular and luminal epithelium at 2.5 and 3.5 dpc. The GPR64 proteins were also weakly detected in the stromal cells at 2.5 and 3.5 dpc and then markedly increased in the stromal cells at 4.5 dpc. Interestingly, GPR64 was remarkably strong in primary decidual cells at implantation sites of 5.5 dpc. The expression of GPR64 proteins in primary decidual cells was changed to the secondary decidual zone (further from the embryo) from the primary decidual zone (closer to the embryo) at 7.5 dpc. These results suggest that GPR64 may have an important role for implantation and decidualization during early pregnancy. To confirm the antibody specificity, we performed immunohistochemistry analysis of GPR64 as a positive control^30, 31^ in mouse epididymal tissue. The expression of GPR64 was detected in apical membranes as well as in some nuclei of epididymal duct epithelial cells. Additionally, the IgG antibody was used as a negative control for immunohistochemistry analysis in the mouse epididymal tissue (Fig. S1).

### Regulation of Gpr64 expression by P4 and E2 in the mouse uterus

**Figure 2:**
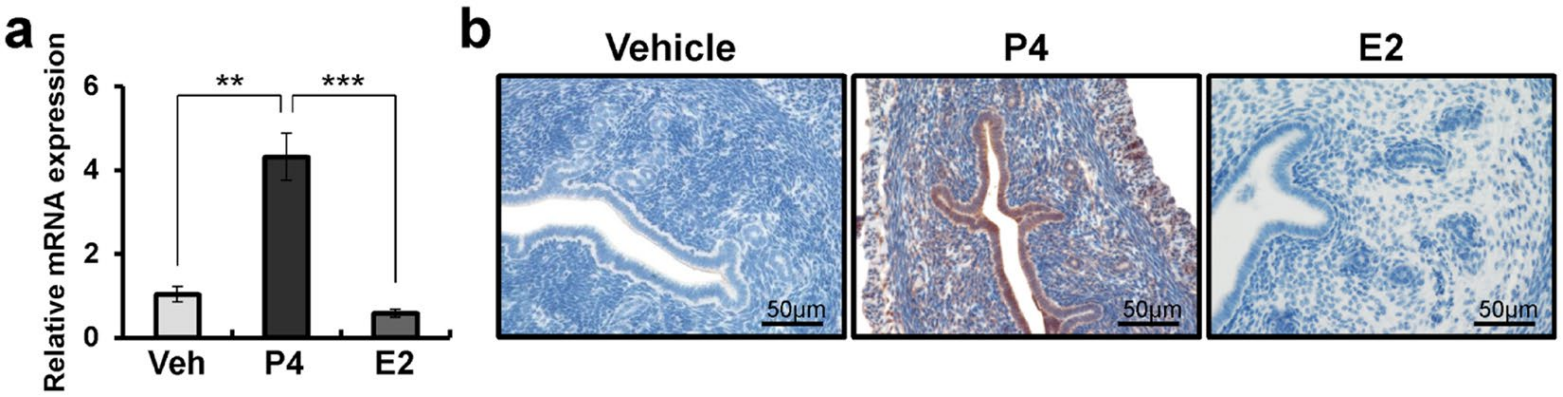
The regulation of *Gpr64* by steroid hormones. (**a**) The expression levels of *Gpr64* from vehicle, progesterone (P4), or estrogen (E2) treated uteri by quantitative real-time PCR. Total RNA used for the real-time PCR assays was prepared from ovariectomized wild-type C57BL/6 mice treated with vehicle, P4, or E2 for 6 hours. The results represent the mean ± SEM of three independent RNA sets. *p < 0.5 and **p < 0.01. (**b**) The immunohistochemistry analysis of GPR64 in mice uteri treated with vehicle, P4, or E2 for 6 hours. Nuclei were counterstained with hematoxylin.

During early pregnancy, P4 and temporal E2 induction are important for embryo implantation^32^. On the basis of Fig. 1 results, we postulated that GRP64 expression is hormonally regulated in the uterus. To investigate whether steroid hormone regulates the expression of *Gpr64*, we treated ovariectomized C57BL/6 female mice with vehicle (sesame oil), P4 (1 mg/mouse), or E2 (0.1 μg/mouse). After 6 hours, we analyzed the expression levels of *Gpr64* mRNA in these murine uteri by real-time PCR. Our results showed that *Gpr64* mRNA expression was significantly increased in the uteri of mice treated with P4 as compared with vehicle and E2 (Fig. 2a). Next, we performed immunohistochemical analysis to identify the spatial expression of the GPR64 expression. These results showed that GPR64 was highly expressed in both stromal and epithelial cells of the uteri treated with P4 for 6 hours compared with other groups (Fig. 2b). Taken together, these finding suggest that P4 induces the *Gpr64* expression but E2 cannot regulate *Gpr64* expression in uteri.

### Gpr64 as a target gene of progesterone receptor in the mouse uterus

**Figure 3:**
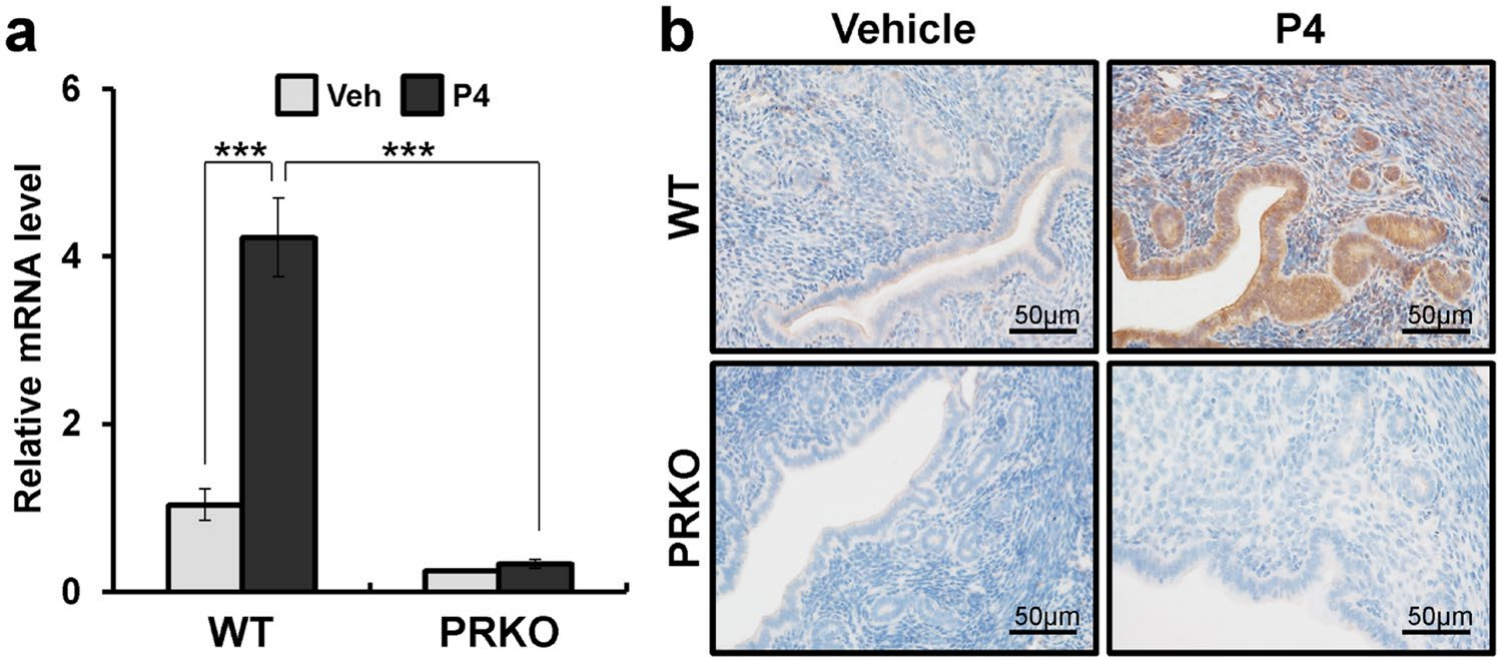
The expression of *Gpr64* in PRKO mice uteri by P4 signal. (**a**) The expression levels of *Gpr64* from P4 treated wild-type or PRKO uteri by quantitative real-time PCR. Total RNA used for the quantitative real-time PCR was prepared from wild-type or PRKO uteri treated with vehicle or P4 for 6 hours. The results represent the mean ± SEM of three independent RNA sets. ***p < 0.001. (**b**) The immunohistochemistry analysis of GPR64 in vehicle or P4-treated uteri. Uterine sections were collected from vehicle or P4 treated wild-type and PRKO mice for 6 hours. Nuclei were counterstained with hematoxylin.

To determine whether *Gpr64* is a P4-PGR signaling targeted gene, we performed real-time PCR in the uterine samples of ovariectomized wild-type and progesterone receptor knock-out (PRKO) female mice treated with vehicle or P4 for 6 hours. As shown in Fig. 3a, levels of *Gpr64* mRNA were significantly increased in the wild-type mice uteri treated with P4 compared with vehicle. However, *Gpr64* mRNA was not increased by P4 treatment in the PRKO mice. To analyze the spatial expression of GPR64 by P4 treatment in the uterus, we performed immunohistochemistry analysis in the vehicle or P4-treated wild-type and PRKO mice. Consistent with the real-time PCR outcomes, we observed GPR64 expression in the stromal and epithelial cells of the uterus section obtained from P4-treated wild-type uterus (Fig. 3b). The GPR64 protein was not detected in the PRKO uterus treated with vehicle or P4.

**Figure 4:**
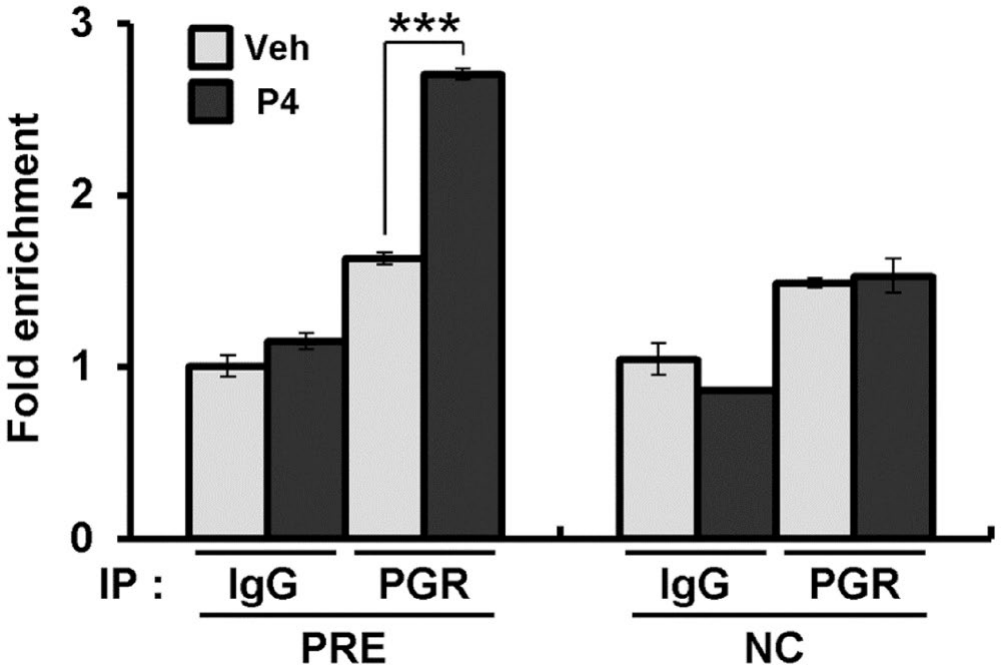
Recruitment of PGR on PRE of *Gpr64* gene. ChIP assay performed with uterine chromatin isolated from C57BL/6 female mice treated with vehicle or P4 for 2 hours using PGR antibody followed by quantitative real-time PCR. A normal rabbit IgG antibody was used as the negative control. NC region on the *Gpr64* gene was used as negative control of ChIP assay. The results represent the mean ± SEM. ***p < 0.001.

To determine whether PGR directly regulates transcriptional activation of *Gpr64*, we performed ChIP analysis on mice uterine chromatin treated with vehicle or P4 for 2 hours based on previous PGR ChIP-seq data^33^. Recruitments of PGR on progesterone response element (PRE; GAATAAAATGATC) were significantly increased by treatment of P4 compared to vehicle (Fig. 4). However, PGR binding was not changed on the negative control region of exon 15 by P4 treatment. These results indicate that *Gpr64* is a direct transcriptional PGR target gene in the uterus.

### Role of Gpr64 in human endometrial stromal cells (hESCs) during ***in vitro*** decidualization

**Figure 5:**
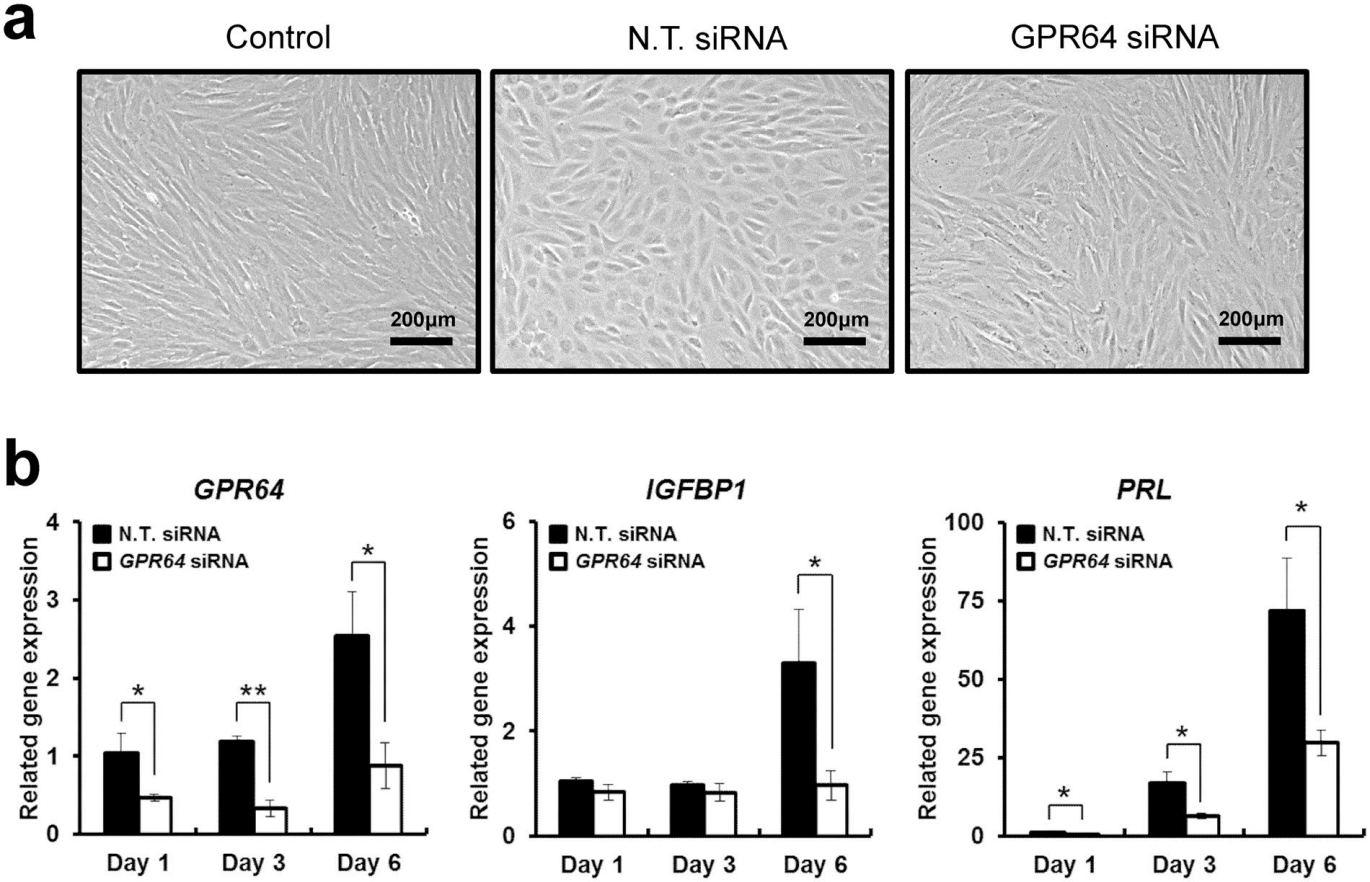
Effect of *GPR64* knock-down during *in vitro* decidualization of hESCs. (**a**) Morphological change of hESCs treated with or without GPR64 siRNA after *in vitro* decidualization treatment for 6 days. Control is non-treatment with *in vitro* decidualization to hESCs. (**b**) The expression levels of *GPR64* were examined on hESCs treated with or without *GPR64* siRNA during *in vitro* decidualization using quantitative real-time PCR. Expression of decidualization marker genes, *IGFPB1* and *PRL* were examined during *in vitro* decidualization of hESCs treated with or without *GPR64* siRNA. The results represent the mean ± SEM. *p < 0.05. The results represent the mean ± SEM. *p < 0.05 and **p < 0.01.

To examine the role of GPR64 in decidualization, we used a well-characterized *in vitro* decidualization model in hESCs^34^. The hESCs were treated with E2, MPA and cAMP to induce decidualization. Prior to treatment, hESCs possessed a fibroblast-like morphology. After *in vitro* decidualization treatment, hESCs enlarged and became round in shape, typical of the decidual transformation (Fig. 5a). Quantitative PCR analysis revealed significantly increased expression levels of the decidualization marker genes (*IGFBP1*and *PRL*) after the decidualization induction (Fig. 5b). To examine GPR64 levels during decidualization process, qPCR analysis was performed on hormone-treated hESCs on day 0, 1, 3 and 6 of *in vitro* decidualization. The expression of GPR64 was increased on day 6 compared to day 1 and day 3.

To further analyze the role of GPR64 in hESC decidualization, we performed siRNA-mediated knockdown of *GPR64* expression. GPR64 attenuation was confirmed that the *GPR64* mRNA levels were significantly decreased in hESCs treated with *GPR64* siRNA compared with non-targeting pool siRNA during *in vitro* decidualization by quantitative real-time PCR (Fig. 5b). hESCs treated with *GPR64* siRNA showed fibroblast-like morphology (Fig. 5a). Quantitative PCR analysis showed that the expression of decidualization marker genes, insulin-like growth factor-binding protein 1 (*IGFBP1*) and prolactin (*PRL*), were significantly reduced in hESCs treated with *GPR64* siRNA as compared to controls (Fig. 5b).

**Figure 6:**
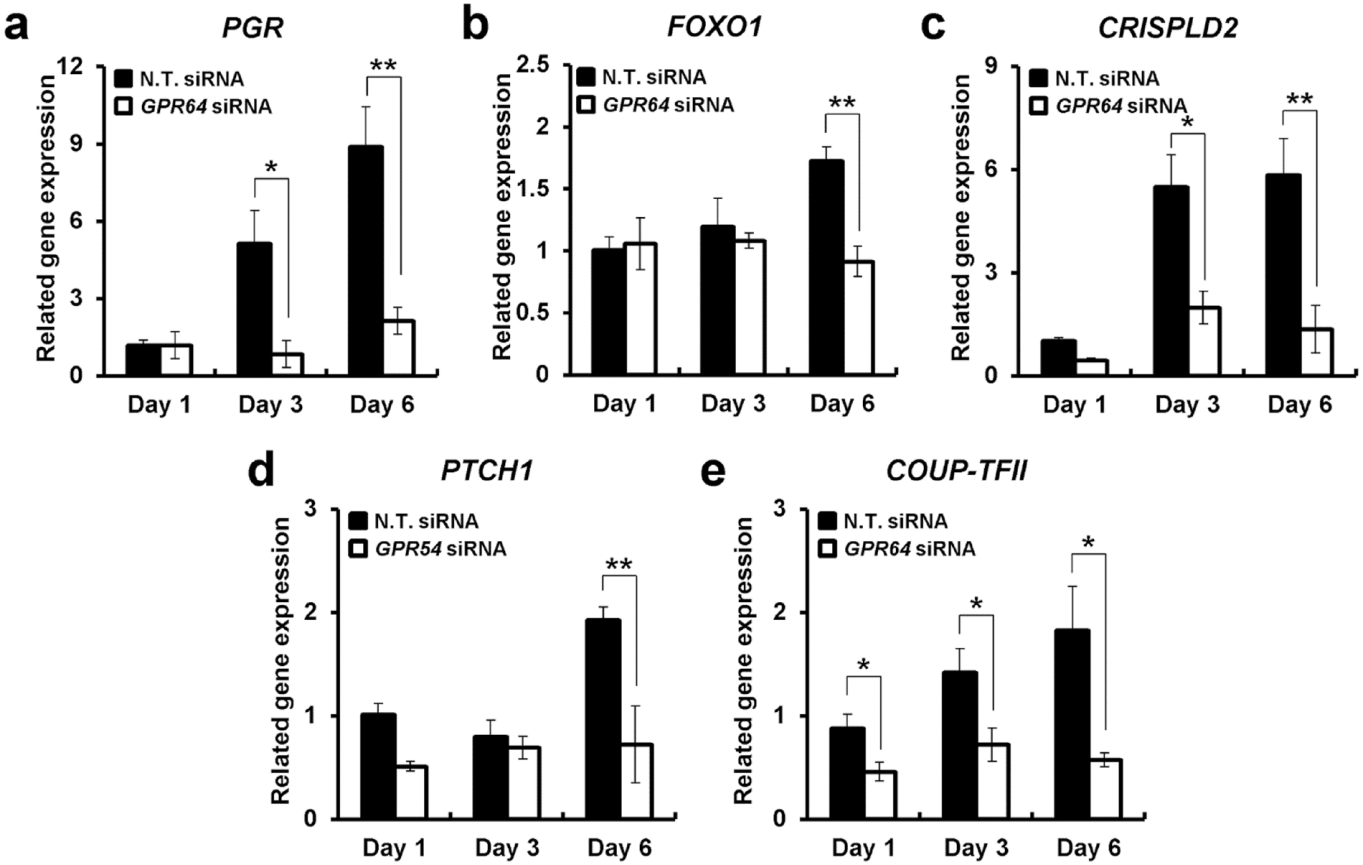
The expression of PGR target genes by reduction of *GPR64* during *in vitro* decidualization of hESCs. The expression levels of *PGR* (**a**), *FOXO1* (**b**), *CRISPLD2* (**c**), *PTCH1* (**d**), and *COUP-TFII* (**e**) during *in vitro* decidualization of hESCs treated with or without *GPR64* siRNA by quantitative real-time PCR. The results represent the mean ± SEM. *p < 0.05 and **p < 0.01.

PGR signaling is a critical regulator of reproductive events associated with endometrial stromal cell decidualization and the maintenance of pregnancy. Therefore, we examined the expression of PGR target genes in decidualized hESC transfected with or without *GPR64* siRNA by real time PCR. The levels of *PGR*, Forkhead box protein O1 (*FOXO1*), cysteine-rich secretory protein LCCL domain-containing 2 (*CRISPLD2*), patched-1 (*PTCH1*), and chicken ovalbumin upstream promoter-transcription factor II (*COUP-TFII*) were increased in hESCs during *in vitro* decidualization. However, the transcriptional induction of *PGR*, *FOXO1*, CRISPLD2, *PTCH1*, and *COUP-TFII* were significantly decreased in hESCs treated with *GPR64* siRNA (Fig. 6). These results suggest that GPR64 plays an important role as a PGR target gene in decidualization.

### Activation of serum response element (SRE) by GPR64 in hESCs

**Figure 7:**
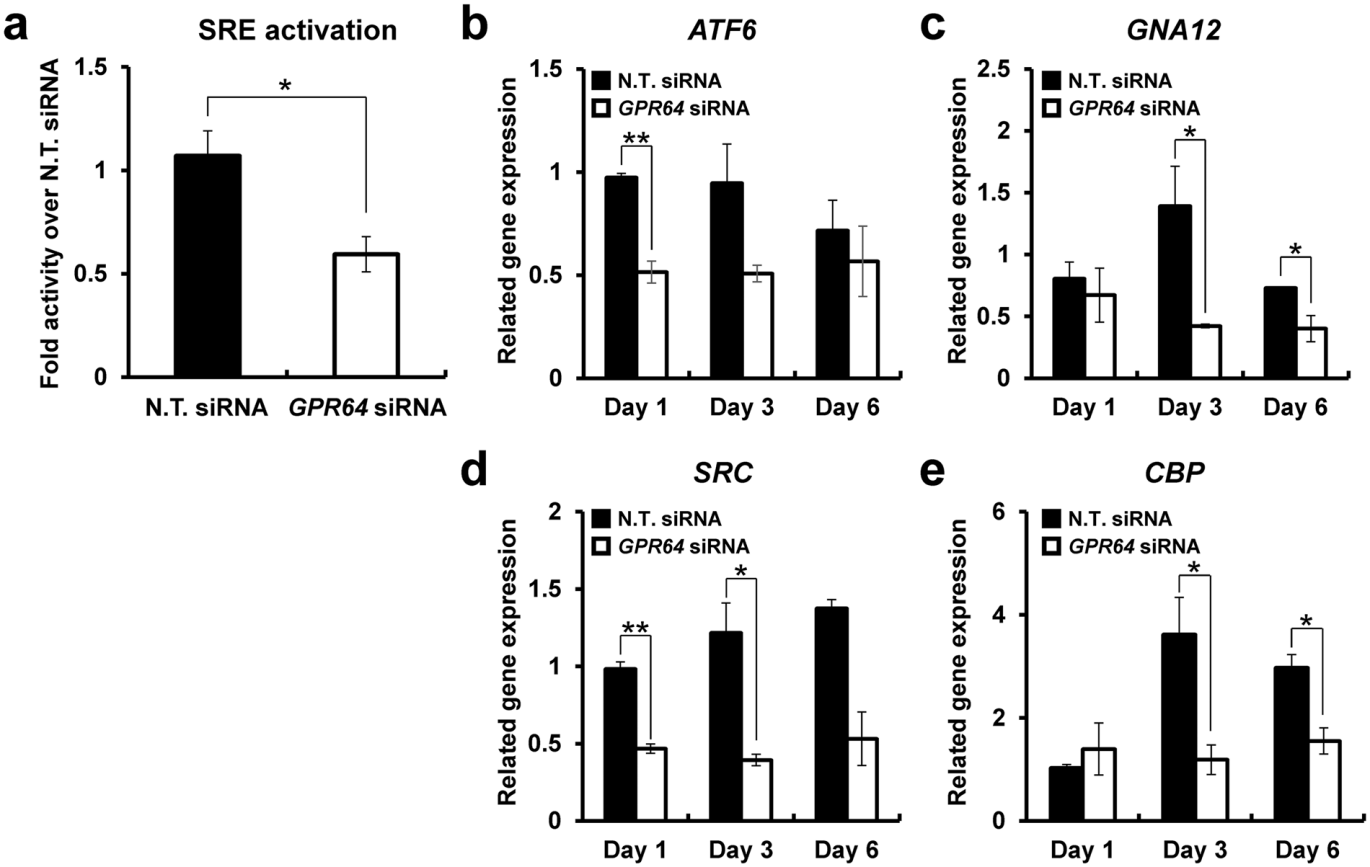
The SRE activation and SRE activated related gene expression is dependent on GPR64 during *in vitro* decidualization of hESCs. (**a**) The activation level of SRE during *in vitro* decidualization of hESCs transfected with or without *GPR64* siRNA. The expression level of related SRE activation genes, *ATF-6* (**b**), *GNA12* (**c**), *SRC* (**d**), and *CBP* (**d**) in decidulized hESCs with or without *GPR64* siRNA transfection. The results represent the mean ± SEM. *p < 0.05 and **p < 0.01.

GPR64 activates serum response element (SRE) for cell adhesion and migration in HEK293 cells^35^. To examine the activity of GPR64 in hESC, we performed SRE-luciferase assay on hESC with and without *GPR64* siRNA. The SRE activation level was significantly decreased in hESCs treated with *GPR64* siRNA compared with non-targeting pool siRNA (Fig. 7a). Serum response factor (SRF) is a transcription factors and binds to SRE in the promoter region of target genes. Therefore, we examined the expression of SRF signaling genes in hESCs with GPR64 deficiency. Our qPCR results revealed that the expression of activating transcription factor-6 (*ATF-6*), G-protein subunit alpha 12 (*GNA12*), *SRC*, and CREB binding protein (*CBP*) were significantly reduced in hESC transfected with *GPR64* siRNA compared to the control (Fig. 7b–e). These results suggest that GPR64 induces SRF signaling molecules to regulate SRE activation.

## Discussion

In this study, we have identified *Gpr64* as a P4-PGR target gene in the mouse uterus. P4 is a well-known critical regulator of the female reproductive system associated with embryo implantation, decidualization and the maintenance of pregnancy^36–39^. The binding between P4 and PGR results in nuclear translocation of PGR and subsequent regulation of P4 target gene transcription. To identify uterine PGR-regulated mechanisms and downstream targets, Jeong *et al*. and Rubel *et al*. performed high density DNA microarray analysis^7^ and chromatin immunoprecipitation followed by deep sequencing (ChIP-Seq)^33^, respectively. These microarray results also showed a *Gpr64* as a potential P4-PGR target gene and ChIP-Seq results also revealed that PGR directly binds to the in gene region of *Gpr64* gene in uteri of ovariectomized mice treated with P4. However, the results of microarray and ChIP-seq have not been examined by qPCR and ChIP analysis.

P4 is required throughout pregnancy associated with embryo implantation, decidualization and the maintenance of pregnancy. In previous studies, *Pgr* gene null mutated female mice show several reproductive defects such as infertility and malfunction of ovulation, implantation, and decidualization^5–7^. The expression levels of PGR increase from 0.5 days post coitum (dpc) and reach peak on 2.5 dpc in the epithelium of mouse uterus^40, 41^. Then, PGR expressions are higher in the primary decidual stromal cells surrounding the embryo at 5.5 dpc of mouse uterus and its expressions move outward into the secondary decidual stromal cells at 7.5 dpc^42–44^. The expression of downstream P4 target genes are important for embryo implantation and decidualization of the uterus such as indian hedgehog (*IHH*), patched-1 (*PTCH1*), hedgehog interacting protein-1 (*HIP-1*), chicken ovalbumin upstream promoter-transcription factor II (*COUP-TFll*), GATA Binding Protein 2 (*GATA2*), cysteine-rich secretory protein LCCL domain-containing 2 (*CRISPLD2*) and are triggered in turn according to the expression pattern of PGR^44–46^. As our results suggest, the mRNA expression of *Gpr64* was gradually increased until 7.5 dpc. GPR64 protein levels were highly expressed in the glandular epithelium at 2.5 dpc, which correlates with elevated P4 levels^42, 43^. Levels of GPR64 were highly expressed in the stromal cells at 4.5 dpc. Those are the time points during which the uterine epithelium prepares for and permits the embryo to implant. GPR64 was highly detected in fully differentiated decidual cells at 5.5 dpc and in the secondary decidual zone (further from the embryo) at 7.5 dpc. Therefore, these results suggest that GPR64 may have an important role both for receptivity in the epithelium and for endometrial decidualization in the stromal cells.

According to previous studies, an acute dose of intraperitoneally administered P4 induces up- and down regulation of P4 target genes in the uterus of ovariectomized mice, and that this induction is lacking in PRKO mice^7, 44, 46, 47^. Our study herein has shown that the mRNA expression levels of *Gpr64* were significantly increased in the ovariectomized mice uteri treated with P4 as compared to vehicle for 6 hours. Additionally, GPR64 was detected highly in the epithelium compartment of the mouse uterus. E2 as well as P4 is important for maintenance of the female reproductive system^48^. However, our results showed that mRNA and protein levels of GPR64 were not affected by E2 stimulation. Additionally, the mRNA and protein levels of GPR64 were significantly upregulated by P4 in wild-type mice, but not in PRKO. Furthermore, our ChIP analysis shows that PGR directly bind to the PRE of *Gpr64* gene. Thus, our results suggest that *Gpr64* may play an important role during early pregnancy as a PGR target.

We showed that the inhibition of *GPR64* by siRNA treatment impaired decidualization capacity in human endometrial stromal cells (hESCs) as evidenced by suppression of *PRL* and *IGFBP1* expression which are known decidualization marker genes. *Gpr64* knockout mice results in male infertility^29^. Mutant mice reveal a dysregulation of fluid reabsorbtion within the efferent ductules, leading to a backup of fluid accumulation in the testis and a subsequent stasis of spermatozoa within the efferent ducts^29^. However, the *Gpr64* knockout female mice are fertile and do not have apparent developmental or behavioral abnormalities. These result suggests that ablation of GPR64 is not enough to abolish decidualization due to functional redundancy or compensation. We postulated that the identification of the regulatory pathways mediated by *Gpr64* would shed light on how these proteins mediate decidualization in the uterus.

P4 is closely associated with endometrial stromal cell decidualization^49^. hESC from patients with endometriosis, with P4-resistance^50^ and PRKO mice demonstrate a decidualization defect, supporting a serious role for P4-PGR signaling in decidualization in both humans and mice^5^. Previous studies have shown that P4 activates IHH and PTCH1 signaling to induce expression of COUP-TFII during decidualization in endometrial stromal cells of mice uteri^51, 52^. COUP-TFII, has been shown to promote decidualization of hESCs via induction of bone morphogenetic protein 2 (BMP2) and inhibition of ESR1 activation^51^. FOXO1 is critical for interferon regulatory factor member 4 (IRF4) expression via binding with PGR on IRF4 gene and a novel transcriptional regulator of endometrial stromal decidualization^53^. Additionally, *CRISPLD2* is a target gene regulated by P4-PGR response and it has critical role during *in vitro* decidualization of hESCs^46^. Our study shows that expression of *PGR*, *FOXO1*, *CRISPLD2*, *PTCH1*, and *COUP-TFII* were significantly decreased in *GPR64-*deficient hESCs.

GPR64 is specifically expressed within the efferent ductules and the initial segment of the epididymis, ductal systems involved in spermatozoon maturation^29^. It is co-localized apical and subapical F-actin in male excurrent duct epithelia^31^. Our results showed that GPR64 is critical as a PGR target gene for decidualization. These functional differences suggested that GPR64 has tissue-specific roles in male and female reproductive function.

GPRs are the largest family of membrane protein involved in signal transduction with heterotrimeric G protein. Heterotrimeric G proteins are classified into four G alpha subunit, Gs, Gi, Gq, and G12^54^. This G alpha subunit dissociates from βγ dimeric subunit, and initiates signal transduction for target gene transcription by various response element such as cAMP response element (CRE), serum response element (SRE), nuclear factor of activated T-cell response element (NFAT-RE), and serum response factor response element (SRF-RE)^54^. Our SRE-luciferase assay and qPCR analysis of SRF signaling genes showed that GRP64 regulates SRE activation in hESC during *in vitro* decidualization. ATF-6 is membrane-bound transcription factor that activated by endoplasmic reticulum stress response^55^. ATF-6 was interacted with SRF for target gene expression by SRE activation^55^. GNA12 is part of G alpha subunit and regulates a variety of cellular responses including activation of Jun N-terminal kinase^56^ and SRE^57^. GNA12 mediated signaling is related to the Rho-family of small GTPase (Rho, Tan, and Cdc42) which regulates cellular activities such as target gene expression and controlling actin cytoskeleton^57^. SRC is a critical factor of decidualization in the SRC deficient mice study^58^. SRC also bind to SRF for co-activate the SRE-mediated transactivation of CBP^59^. CBP activates transcription that interacted with transcription factor managed by cAMP response element binding protein (CREB) domain, and had a SRE in promoter region regulated by SRC-SRF^59^. Therefore, our results suggest that GPR64 regulates SRE activation through SRF-related transcription factors.

In summary, we first addressed that the *Gpr64* is a target gene of P4-PGR signaling in the uterus. Inhibition of *GPR64* by siRNA-mediated knockdown suppresses decidualization of hESCs. Attenuation of *GPR64* decreased the expression of *PGR*, *FOXO1*, *CRISPLD2*, *PTCH1*, and *COUP-TFII* in hESCs during *in vitro* decidualization. These results suggest that *Gpr64* plays an important role for successful decidualization and is a target of P4-PGR signaling in mice as well as humans.

## Methods

### Animals and tissue collection

Mice were cared for and used in the designated animal care facility according to Michigan State University’s institutional guidelines. All animal procedures were approved by the Institutional Animal Care and Use Committee of Michigan State University. For the early pregnancy study, wild-type C57BL/6 female mice at 8 weeks of age were mated with wild-type C57BL/6 male mice and uterine samples from pregnant mice were obtained at different days of pregnancy. The morning of vaginal plug observation was designated as 0.5 days post coitum (dpc) (n = 3). For the study of steroid hormone regulation, wild-type and PRKO mice^5^ at 6 weeks of age were ovariectomized. Two weeks post-surgery, ovariectomized mice were injected with vehicle (sesame oil; Veh), P4 (1 mg/mouse), and estradiol (0.1 μg/mouse). Mice were euthanized at 6 hours after injection (n = 3 per genotype per treatment). Uterine tissues were immediately frozen at the time of dissection and stored at −80 °C for RNA extraction or fixed with 4% (v/v) paraformaldehyde for immunohistochemistry.

### RNA isolation and quantitative real-time PCR

Total RNA was extracted using the Trizol reagent (Invitrogen, Carlsbad, CA). cDNA was produced from 1 μg of total RNA using random hexamers and MMLV Reverse Transcriptase (Invitrogen Corp., Carlsbad, CA). Real-time PCR was performed using the real-time PCR SYBR Green detection system (Bio-Rad, Hercules, CA) according to the manufacturer’s instructions (PE Applied Biosystems, Foster City, CA). mRNA quantities were normalized against the housekeeping gene, 18 S RNA. The sequences of the primers used for mouse *Gpr64* were 5′-GCCCTTCCTCACCAGAAGAG-3′ and 5′-ATAAGGGCATGATCAAGGGG-3′, human *GPR64* were 5′-CTGCAGGATCCCATTGTCTG-3′ and 5′-TGAAAGGGGTTGAATCTCCC-3′, for *IGFBP1* were 5′-CTATGATGGCTCGAAGGCTC-3′ and 5′-TTC TTGTTGCAGTTTGGCAG-3′, for *PRL* were 5′-CATCAACAGCTGCCACACTT-3′ and 5′-C GTTTGGTTTGCTCCTCAAT-3′, for *PTCH1* were 5′-TGTGCGCTGTCTTCCTTCTG-3′ and 5′-ACGGCACTGAGCTTGATTC-3′, for *CRISPLD2* were 5′-CGGACGAGATGAATGAGGTG-3′ and 5′-TGACCGCAGAGGTTTTCTTG-3′, for *ATF-6* were 5′-GCCTTTATTGCTTCCAGCAG-3′ and 5′-TGAGACAGCAAAACCGTCTG-3′, for *GNA12* were 5′-ATGGTCTCCTCCAGCGAGTA-3′ and 5′-CTTGATGCTCACGGTCTTCA-3′, for *SRC* were 5′-AGGGGAGTTTGCTGGACTTT-3′ and 5′-AGGTTCTCTCCCACCAGGAT-3′, for *CBP* were 5′-GAATGCCGTACCCTACTCCA-3′ and 5′-GGCTGTCCAAATGGACTTGT-3′, and for 18S were 5′-GTAACCCGTTGAACCCCATT-3′ and 5′-CCATCCAATCGGTAGTAGCG-3′.

### Immunohistochemistry

Immunohistochemistry analysis was performed as previously described^60^. Uterine cross sections from paraffin-embedded tissue were cut into 6 μm sections, mounted on silane-coated slides (12-550-15, Fisher Scientific, Pittsburgh, PA), deparaffinized and rehydrated in a graded alcohol series. Sections were pre-incubated with 10% normal rabbit serum in phosphate-buffered saline (PBS; pH 7.5) and then incubated with anti-GPR64 (1:500 dilution, sc-69492, Santa Cruz, Santa Cruz, CA) antibody in PBS supplemented with 10% normal goat serum overnight at 4 °C. The next day, sections were washed with PBS and incubated with secondary antibody conjugated to horseradish peroxidase (Vector Laboratories, Burlingame, CA) for 1 hour at room temperature. Immunoreactivity was detected using diaminobenzidine (DAB-Vector Laboratories, Burlingame, CA) then counterstained with hematoxylin and coverslipped with permount. Imuunostaining was analyzed using microscopy software from NIS Elements, Inc. (Nikon, Melville, NY).

### Chromatinimmunoprecipitation (ChIP)

ChIP was performed as previously described^61^. Briefly, ovariectomized mice were injected with vehicle (sesame oil) or P4 (1 mg/mouse) after two weeks post-surgery. Uteri were removed from euthanized mice at 2 hours after injection (n = 10 per treatment). For each ChIP reaction, 100 μg of chromatin was immunoprecipitated by 4 μg of antibodies against PGR (sc7208; Santa Cruz Biotechnology, Santa Cruz, CA). Eluted DNA was amplified with specific primers using SYBR Green Supermix (Bio-Rad Laboratories, Inc., Hercules, CA). Primers used in PCR were as follows: PRE (forward: 5′-GGGGACTCCTTTTTGGTGGA-3′; reverse: 5′-TCAGAAGCCACCAGACCGTG-3′) and negative control (NC) (forward: 5′-GCCCACCGTTATCGTCTTCC-3′; reverse: 5′-CAGGGGGATCGTAGGCTGAG-3′). The resulting signals were normalized to input activity.

### Human endometrial stromal cell culture and *in vitro* decidualization

We used previously isolated Human endometrial stromal cells (hESCs) for this study^34, 62^. hESCs were then maintained in phenol red–free RPMI-1640 medium (Gibco, Grand Island, NY) containing 0.1 mM sodium pyruvate (Gibco, Grand Island, NY), 10% fetal bovine serum (FBS; Gibco, Grand Island, NY) depleted of steroids by pre-treatment with dextran-coated charcoal (Sigma Aldrich, St. Louis, MO) (Charcoal-stripped FBS; CS-FBS), and 1% penicillin streptomycin (P/S; Gibco, Grand Island, NY). Cells were cultured in monolayer at 37 °C in 5% CO_2_. The induction of *in vitro* decidualization has been previously described^46, 62^. To induce *in vitro* decidualization, cells were washed with PBS and incubated to OPTI-MEM medium (Gibco, Grand Island, NY) containing 2% CS-FBS, 10 nM estradiol (E2; Sigma-Aldrich, St. Louis, MO), 1 mM medroxyprogesterone acetate (MPA; Sigma-Aldrich, St. Louis, MO), 50 μM cAMP (Sigma-Aldrich, St. Louis, MO), and 1% P/S. Differentiation medium was changed every 48 hours for a total of 6 days. For *GPR64* knockdown, small interfering RNA (siRNA) was obtained from Dharmacon (Lafayette, CO). Human *GPR64* siRNA was transfected using Lipofectamine 2000 reagent (Invitrogen Crop., Carlsbad, CA) prior to *in vitro* decidualization.

### SRE-luciferase assay

hESCs were were transiently co-transfected with 1 ug/well of the cis-reporter plasmids pSRE-luc (PathDetect, La Jolla, CA) and 100 ng/well pRL-TK (Promega, Madidon, WI) with or without *GPR64* siRNA for 24 hours in 24 well culture dish. After 24 hour, transfection medium was replaced with low serum growth medium (0.5% FBS). The assay was terminated 30 hour post transfection medium change. Luminescence were determined using Dual-Luciferase reporter assay system reagent kit (Promega, Madidon, WI) according to the manufacturer’s instructions and measured using victor 3 multiabel plate reader (PerkinElmer, Groningen, The Netherlands).

### Statistical analysis

Statistical analyses were performed with the Student’s t-test for data with two groups. For data containing more than two groups, we performed analysis of variance (ANOVA) test and analyzed by Tukey or Bonferroni test for pairwise t-test. All data are presented as means ± SEM. p < 0.05 was considered statistically significant. All statistical analyses were performed using the Instat package from GraphPad (San Diego, CA, USA).

## Electronic supplementary material

Fig. S1. Histological analysis confirmed the specificity of GPR64 antibody in in mouse epididymis tissue.
