# Supplementary material for: G-protein coupled receptor 64 is required for decidualization of endometrial stromal cells
Source: Sci Rep. 2017 Jul 10;7:5021. doi: 10.1038/s41598-017-05165-8 (PMC5503986; doi:10.1038/s41598-017-05165-8)
Supplement: Supplementary file 1 — Fig. S1. Histological analysis confirmed the specificity of GPR64 antibody in in mouse epididymis tissue. [file 41598_2017_5165_MOESM1_ESM.pdf]

**G-protein coupled receptor 64 is required for decidualization of endometrial stromal cells.**

Jung-Yoon Yoo[1,†], Jong Il Ahn[2,3,†],Tae Hoon Kim[1], Sungryul Yu[4], Ji Yeon Ahn[2,3], Jeong Mook Lim[2,3,*], and Jae-Wook Jeong[1,5,*]

[1]Deparment of Obstetrics and Gynecology & Reproductive Biology, Michigan State University, College of Human Medicine, Grand Rapid, MI 49503, United State
[2]Laboratory of Stem Cell and Bioevaluation, Major in Biomodulation, Seoul National University, Seoul 08826, Republic of Korea
[3]Department of Agricultural Biotechnology, Seoul National University, Seoul 08826, Republic of Korea
[4]Department of Clinical Laboratory Science, Semyung University, Jecheon 27136, Republic of Korea
[5]Department of Women's Health, Spectrum Health System, Grand Rapids, MI 49341, United State

[†] These authors contributed equally to this study.

*Correspondence to;
Jeong Mook Lim, Ph.D.
Laboratory of Stem Cell and Bioevaluation, Major in Biomodulation
College of Agriculture and Life Science, Seoul National University
Daehak ro-1, Seoul 151-921, Republic of Korea
Phone: +82-2-880-4806
Fax: +82-2-874-2555
E-mail: limjm@snu.ac.kr

Jae-Wook Jeong, Ph.D.
Department of Obstetrics, Gynecology & Reproductive Biology
Michigan State University College of Human Medicine
333 Bostwick Avenue NE, Grand Rapids, MI 49503
Phone: 616-234-0987
Fax: 616-234-0990
E-mail: JaeWook.Jeong@hc.msu.edu

**This PDF include**:
**Supplemental Figure S1.** Histological analysis confirmed the specificity of GPR64 antibody in in mouse epididymis tissue.

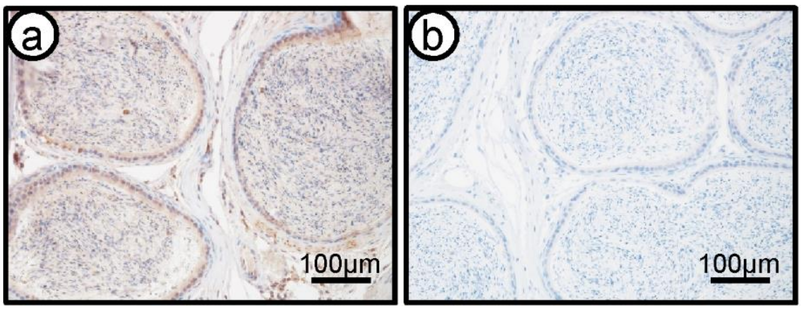


**Fig. S1.** Histological analysis confirmed the specificity of GPR64 antibody in in mouse epididymis tissue. (a) High levels of GPR64 proteins were observed in apical membrane of epithelia. (b) Immunohistochemical analysis of IgG antibody was intended for use as a negative control with GPR64 proteins in the mouse epididymal tissue.
